# Sensitivity to Pulse Phase Duration as a Marker of Neural Health Across Cochlear Implant Recipients and Electrodes

**DOI:** 10.1007/s10162-021-00784-5

**Published:** 2021-02-08

**Authors:** Ning Zhou, Zhen Zhu, Lixue Dong, John Galvin

**Affiliations:** 1 Department of Communication Sciences and Disorders, East Carolina University, Greenville, NC 27834 USA; 2 Department of Engineering, East Carolina University, Greenville, NC 27834 USA; 3 House Ear Institute, 2100 W. Third St., Suite 101, Los Angeles, CA 90057 USA

**Keywords:** cochlear implant;, charge integration efficiency;, leakiness;, neural health;, dynamic range;, across-site variability

## Abstract

In cochlear implants, loudness has been shown to grow more slowly with increasing pulse phase duration (PPD) than with pulse amplitude (PA), possibly due to “leaky” charge integration. This leakiness has been recently quantified in terms of “charge integration efficiency,” defined as the log difference between the PPD dynamic range and PA dynamic range (both expressed in charge units), relative to a common threshold anchor. Such leakiness may differ across electrodes and/or test ears, and may reflect underlying neural health. In this study, we examined the across-site variation of charge integration in recipients of Cochlear© devices. PPD and PA dynamic ranges were measured relative to two threshold anchors with either a 25- or 50-microsecond PPD. Strength-duration functions, previously shown to relate to survival of spiral ganglion cells and peripheral processes, were compared to charge integration efficiency on selected electrodes. Results showed no significant or systematic relationship between the across-site variation in charge integration efficiency and electrode position or threshold levels. Charge integration efficiency was poorer with the 50-μs threshold anchor, suggesting that greater leakiness was associated with larger PPD dynamic ranges. Poorer and more variable charge integration efficiency across electrodes was associated with longer duration of any hearing loss, consistent with the idea that poor integration is related to neural degeneration. More variable integration efficiency was also associated with poorer speech recognition performance across test ears. The slopes of the strength-duration functions at maximum acceptable loudness were significantly correlated with charge integration efficiency. However, the strength-duration slopes were not predictive of duration of any hearing loss or speech recognition performance in our participants. As such, charge integration efficiency may be a better candidate to measure leakiness in neural populations across the electrode array, as well as the general health of the auditory nerve in human cochlear implant recipients.

## INTRODUCTION

In cochlear implants, loudness of the electrical pulses can be increased by increasing the pulse amplitude (PA) or pulse phase duration (PPD). However, these two parameters do not trade off equally (Shannon 1985; Zeng et al. 1998; Chatterjee et al. 2000). The same amount of charge distributed over a longer PPD is less effective in producing excitation (e.g., Shannon 1985). This is thought to reflect the leakiness of the neural membrane. Leaky charge integration may reflect neural degeneration associated with hearing loss. Such degeneration begins with the loss of peripheral processes, demyelination of the cell body (in animals), and, ultimately, cell loss (Hardie and Shepherd 1999). These anatomical changes may lead to a reduced number of nodes on a single fiber that falls into the depolarized region (Rattay 1989). Central migration of the spike initiation site to the extensively myelinated large-diameter axon will lead to a shorter integration time constant (van den Honert and Stypulkowski 1984; Shepherd et al. 2001). The leakiness in charge integration may be a useful indicator of neural degeneration across electrodes in implant recipients.

Zhou et al. (2020) described a clinically applicable method to quantify charge integration in the auditory nerve. “Charge integration efficiency” (CIE) was defined as the log difference in dynamic range (DR) as PPD or PA is increased, relative to a common threshold anchor. Note that PPD and PA DRs were quantified in terms of charge (nanocoulombs, or nC) before calculating CIE. Anchoring the two DRs to a common threshold allowed for direct comparison of loudness growth with increasing PPD or PA. Zhou et al. (2020) found that DRs were significantly larger with increasing PPD than with increasing PA. CIE was significantly correlated with duration of profound hearing loss, suggesting a potential link between charge integration and the extent of neural degeneration. In Zhou et al. (2020), CIE was measured on a single middle electrode for all test ears. While leakiness was observed in all test ears, it is unlikely that this single-electrode measure represented the condition of the whole auditory nerve. Previous studies suggest that neural degeneration is patchy, and patterns of patchiness do not systematically vary with etiology of hearing loss (Nadol 1997).

The across-site variance reported in many psychophysical studies is thought to reflect the variability in underlying neural conditions, at least to some extent, but a considerable proportion of across-site variance may be related to electrode position (e.g., Long et al. 2014; Schvartz-Leyzac et al. 2020). For example, for lateral-wall electrodes which presumably create broader excitation, detection thresholds are higher (Long et al. 2014), ECAP growth functions are steeper (Schvartz-Leyzac et al. 2020), sensitivity to amplitude modulation is poorer (Zhou et al. 2018), and sensitivity to stimulation rate is better (Zhou and Pfingst 2016; Zhou et al. 2019). Assuming that electrode position may contribute to loudness growth with increasing PA and PPD, calculating the log difference between the two DRs (as in the CIE measure) may reduce the effect of electrode position to some extent. In the present study, we measured CIE across all available electrodes in cochlear implant recipients that represented a wide range of auditory deprivation before implantation. In Experiment 1 of the present study, CIE was compared to duration of hearing deprivation and to speech understanding in noise. Given the expected non-uniform neural density and health across the electrode array, we expected that there would be substantial across-site variability in CIE. We also predicted that greater across-site variance in CIE and poorer across-site mean in CIE (i.e., degeneration across the cochlea) would be associated with longer hearing deprivation and poorer speech recognition performance.

Another method to quantify the integration property of the auditory nerve is the strength-duration function (Pfingst et al. 1991; Moon et al. 1993; Zeng et al. 1998), where the PA is adjusted to maintain threshold for a range of PPDs. Assuming an equal tradeoff between PA and PPD, the slope of the strength-duration function would be − 6 dB/doubling of PPD, reflecting perfect charge integration over time. Strength-duration functions have been measured psychophysically across species and in single auditory nerve fibers (van den Honert and Stypulkowski 1984; Miller et al. 1999; Parkins and Colombo 1987; Smith and Finley 1997; Moon et al. 1993; Shepherd et al. 2001). The behavioral strength-duration slopes were steeper than those physiologically measured in single fibers (Pfingst et al. 1991), and steeper with broad monopolar stimulation than with focused bipolar stimulation (Smith and Finley 1997; Miller et al. 1999). These differences reflect integration of activity across fibers and are consistent with the idea that cell loss will negatively affect integration across PPD. Importantly, physiological studies reported shorter chronaxie of the strength-duration functions (i.e., poorer integration of PPD) in animals that were long-term deafened or those whose peripheral processes were mechanically removed (van den Honert and Stypulkowski 1984; Shepherd et al. 2001). Prado-Guitierrez et al. (2006) provided further evidence that the effect of PPD on electrically evoked auditory brainstem responses (EABRs) and electrically evoked compound action potential (ECAP) responses in guinea pigs was correlated with neural survival. The authors showed that guinea pigs with poorer neural survival required a smaller PA reduction for longer PPDs to evoke the same neural responses compared with shorter PPDs. Given the evidence from previous behavioral and physiological studies, in Experiment 2, strength-duration functions were compared to CIE at various electrodes. We expected that these strength-duration slopes and CIE would be correlated across electrodes and test ears.

## METHODS

### Participants

Eleven adult cochlear implant ears were tested in this study (3 bilateral implant recipients, 6 unilateral implant recipients). Both ears of bilateral implant recipients S16 and S25 were tested. All participants were postlingually deaf, native English speakers and were recipients of Cochlear© devices (Cochlear Corporation, Englewood, CO), except for S16 who was perilingually deaf. All participants had at least 3 years of implant experience. The mean age at testing was 66.7 years, the mean duration of profound hearing loss was 12.9 years, the mean duration of any hearing loss was 29.9 years, and the mean implant experience was 9.1 years. Demographic information for all test ears is shown in Table 1. All participants provided written informed consent before the experiments began. The study was approved by the East Carolina University Institutional Review Board (UMCIRB 13-001783).

**Table 1:** Demographic information for CI participants. *CI exp* years of experience with CI

Ear	Gender	Age at test (years)	CI exp (years)	Duration profound HL (years)	Duration any HL (years)	Implant	Processor
S1R	M	80.4	11.3	6.0	19.0	CI24RE (CA)	CP1000
S4L	F	60.2	7.8	4.6	22.4	CI24RE (CA)	CP810
S7R	F	73.7	8.5	27.8	34.8	CI24RE (CA)	CP1000
S16L	M	57.7	12.6	45.1	45.1	CI24RE (CA)	CP1000
S16R	M	57.7	10.7	47.0	47.0	CI24RE (CA)	CP1000
S18L	F	67.4	4.7	3.6	33.6	CI422	CP910
S19L	F	72.7	12.0	4.3	44.3	CI24RE (CA)	CP1000
S22R	F	74.5	7.0	0.4	17.4	CI24RE(CA)	CP920
S25L	F	62.2	11.7	0.7	18.7	CI24RE (CA)	CP900
S25R	F	62.2	10.9	1.4	19.4	CI24RE (CA)	CP900
S31L	M	69.7	3.8	1.5	26.5	CI422	Kanso
Mean		67.1	9.2	12.9	29.9		

### Psychophysical Stimuli

Stimuli were 300 ms, charge-balanced, anodic-first, symmetric, biphasic pulse trains. The stimulation rate was 1000 pps. The inter-phase gap was 8 μs and the stimulation mode was monopolar (MP1+2). Note that in this study, PPD values are for each phase of the biphasic pulse. Stimuli were presented via a research interface (NIC II) connected to a Nucleus® Freedom processor (Cochlear Corporation, Englewood, CO) and controlled by MATLAB custom software, allowing precise control of stimulation parameters. The range of PPD values used in the present study was 25–429.8 μs, with 429.8 μs corresponding to the hardware limit of the NIC II system. Also, large PPD values may be difficult to interpret, as very long PPDs may activate multiple spikes in single fibers (Moon et al. 1993).

### Experiment 1: Charge Integration Efficiency (CIE) Across Electrodes

#### Dynamic Range (DR) Estimation

**Fig. 1:**
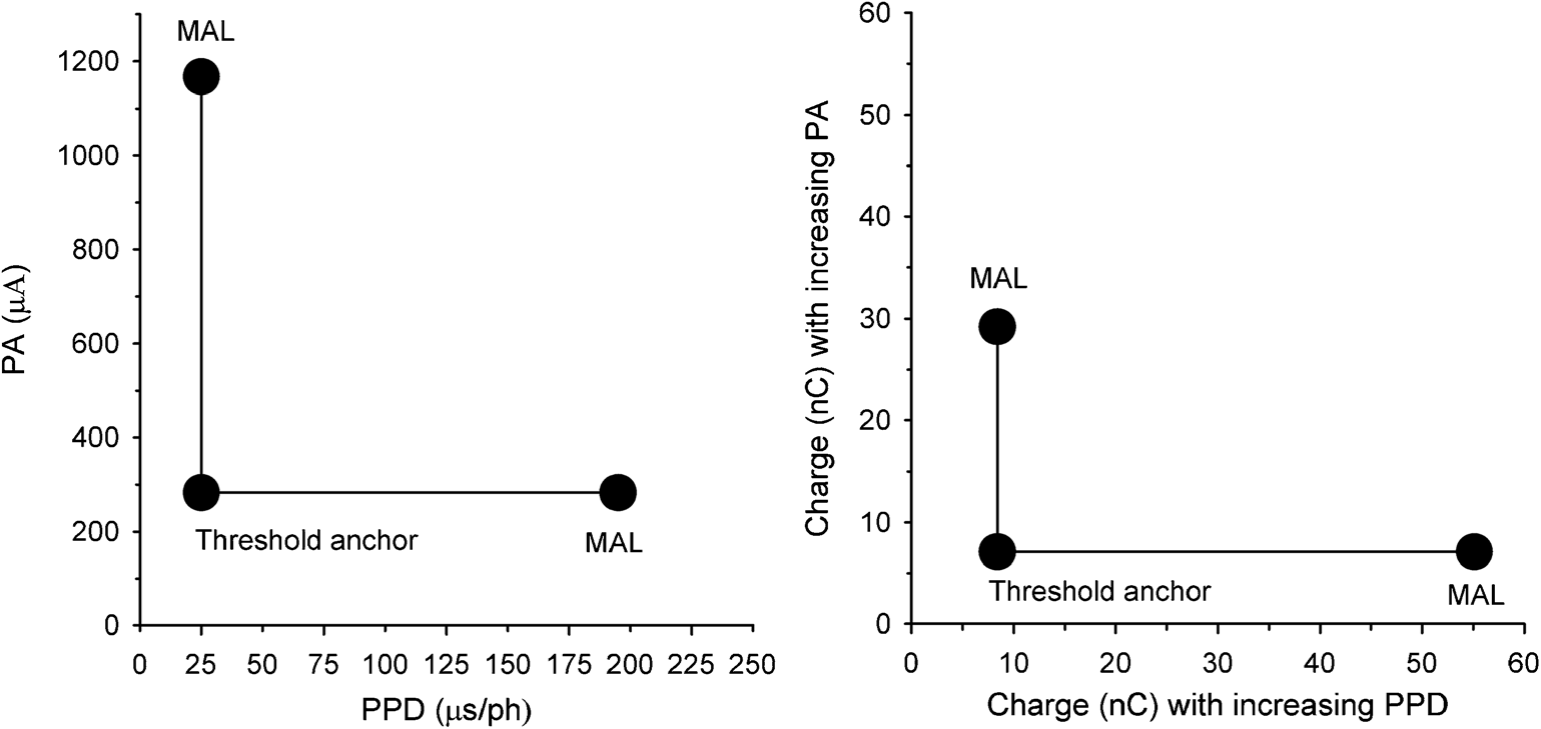
Left panel: Illustration of method used to measure DRs for the PA and PPD stimuli with the 25-μs anchor using data from S16R electrode 10. The DR for the PA stimulus was measured first. The PPD was fixed at 25 μs and the PA was adjusted until obtaining threshold, after which the PA was further increased until achieving maximum acceptable loudness (MAL). Next, the PPD DR was measured. The PA was fixed at the threshold obtained for PA stimulus and the initial PPD was 25 μs; as such, the same threshold anchored the PA and PPD DRs. The PPD was then increased until achieving MAL. Right panel: Threshold and MAL values converted to charge (nC) for the PA and PPD stimuli

DRs, defined as the difference between threshold and maximum acceptable loudness (MAL), were estimated for each test ear and for each electrode that was activated in the clinical speech processor map; DRs were measured in random order across electrodes. For each electrode, DRs were estimated for stimuli in which PA or PPD was increased, relative to a common anchor threshold. The left panel of Fig. 1 illustrates the procedure for estimating PA and PPD DRs, using data from test ear S16R, electrode 10. First, the DR was estimated for the “PA stimulus.” In the Fig. 1 example, the PPD was fixed at 25 μs and the PA was adjusted until obtaining threshold (282 microamps, or μA); PA was then increased from 282 μA until achieving MAL (1167 μA). Next, the DR was measured for the “PPD stimulus.” The PA was fixed at the threshold measured for the PA stimulus (282 μA), and the PPD was increased from 25 μs until reaching MAL (195 μs). Thus, the common threshold anchor for both the PA and PPD DRs was 282 μA and 25 μs. In this study, DRs were measured relative to a 25-μs common threshold anchor (“25-μs anchor” condition) or to a 50-μs common threshold anchor (“50-μs anchor” condition). The 50-μs anchor condition was expected to elicit greater leakiness across electrodes and test ears, due to the longer PPD and larger PPD DRs.

Responses were collected using a graphic user interface, where participants could adjust the stimulus magnitude, either in PA or PPD. The PA could be adjusted in 25, 5, or 1 clinical level (CL) steps, and the PPD could be adjusted in 25, 5 and 1 μs steps. Thresholds were measured using method of adjustment, and MAL was measured using method of limits. When approaching threshold or MAL, participants were instructed to use the smallest step size. Thresholds and MALs were measured at least twice at each available electrode. For the PA stimulus, a third repeat was run if the first two PA thresholds differed by more than 10 CL; the closest two values were averaged as the PA threshold. When measuring the PPD DR, participants were asked to confirm whether the PPD stimulus was just audible at 25 μs (or 50 μs) for the fixed PA using method of adjustment; any adjustments made to PPD threshold were recorded. MAL was repeated if the first two runs differed by more than 5 CL or 10 μs. The closest two values were averaged.

Thresholds, MALs, and DRs for the PA and PPD stimuli were converted to the common unit of electric charge (nC) (see Fig. 1, right panel). CIE was calculated as 20×log (PPD DR_nC_ / PA DR_nC_), as in Zhou et al. (2020; i.e., the difference between the log PPD DR and log PA DR). CIE was calculated for both the 25-μs and 50-μs anchors at each available electrode.

#### Speech Understanding in Noise

Recognition of AzBio sentences (Spahr et al. 2012) was measured in 10-talker babble; the signal to noise ratio (SNR) was 20 dB SNR. Participants were tested while using their clinical speech processors and everyday settings (e.g., ACE strategy, the clinical default stimulation rate of 900 pps/channel, etc.). For unilateral implant recipients, the non-implanted ear was always plugged. For test ears S7 and S18, there was minimal residual acoustic hearing in the non-implanted ear (pure-tone average threshold across 250, 500, and 1000 Hz: 80 dB HL for S7, and 75 dB HL for S18). Given the approximately 20–30 dB of attenuation provided by the ear plug, speech stimuli were not audible in the non-implanted ear. For bilateral implant recipients, the speech processor was removed from the non-test ear. The speech stimuli and background noise were played via a loudspeaker located 1 meter away from the participant’s head at 0° azimuth. All speech testing took place in a double-walled sound attenuated booth. Sentences were presented at 65 dBA. Participants were instructed to repeat as many words in the sentence as they could identify. Scoring was based on the number of correctly identified keys words. Two lists of 20 sentences were presented, and scores were averaged across lists.

### Experiment 2: Strength-Duration Functions

Four electrodes were selected to measure the strength-duration functions. Two electrodes with relatively small PPD DRs (in nC) and two electrodes with relatively large PPD DRs measured with the 50-μs anchor in Experiment 1 were selected for testing. Electrodes were selected with the 50-μs anchor because the longer PPD was assumed to induce greater leakiness than would the 25-μs anchor. PA thresholds and MALs were measured at 5 PPDs: 25, 50, 100, 200, and 400 μs.

To estimate the range of acceptable PA values, PA DRs were first measured for each PPD; thresholds were measured using method of adjustment, and MALs were measured using method of limits, as in Experiment 1. After estimating these DRs, a three-alternative forced-choice (3AFC) adaptive procedure (2-down/1-up) was used to formally measure the PA thresholds, converging on the 79.1 % correct point on the psychometric function (Levitt 1971). During testing, the stimulus was randomly assigned to one of the three intervals; the other two intervals contained no stimuli. For each PPD condition, the initial PA level was set to 50 % of the estimated DR. Participants were asked to select the interval that contained the stimulus. The PA was adjusted according to the correctness of the response. During each trial, the initial step size was 10 CL, and the step size was progressively reduced to 5 CL, 2 CL, and finally, 1 CL, as the number of reversals increased. The final 6 of 12 reversals in PA were averaged as threshold. Thresholds were measured two times for each PPD, and the order of testing was randomized across PPDs and sites. A third repeat was run if the first two PA thresholds differed by more than 10 CL. The closest two values were averaged. MAL data from the initial DR estimation for each PPD condition were used to calculate strength-duration functions at MAL.

Threshold and MAL values were converted to dB (re: 1 μA). For each electrode, the reduction in PA at threshold and MAL was calculated for each PPD doubling (25–50, 50–100, 100–200, and 200–400 μs). Then a linear slope was fit across the entire function (dB/doubling).

### Statistical Analyses

Data for Experiments 1 and 2 were primarily analyzed using linear mixed models (LMMs), rather than repeated-measures analyses of variance, because LMMs could accommodate missing data due to deactivated electrodes in some test ears. Data were also analyzed using stepwise multilinear regressions and Pearson correlations where appropriate; SPSS (Version 24.0; Armonk, NY) was used for all analyses. For all analyses, the significance level was *P* < 0.05.

## RESULTS

### Experiment 1: CIE Across Electrodes

**Fig. 2:**
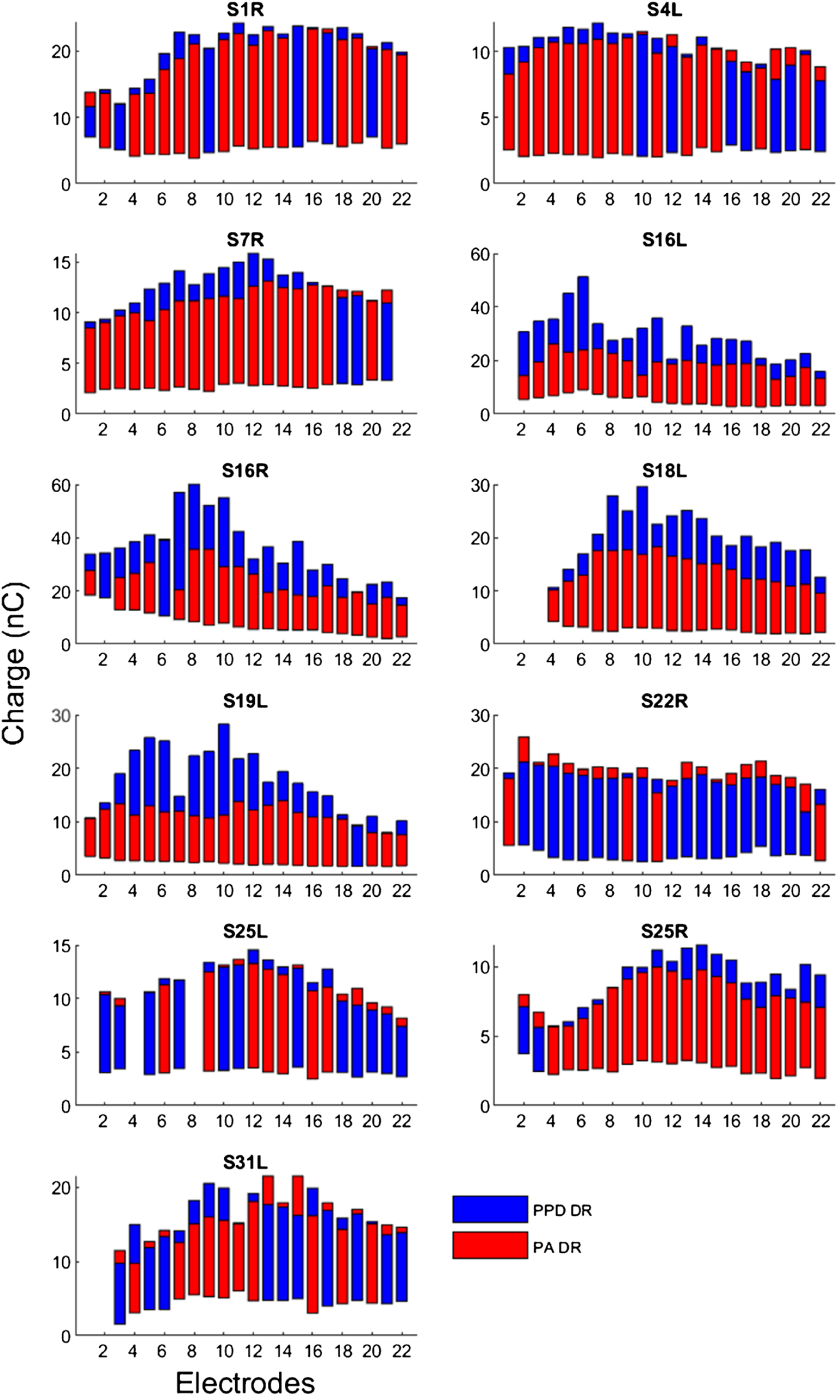
Thresholds and MALs (both in nC) as a function of electrodes for test ears with the 25-μs anchor. The bottom of each bar shows threshold, and the top of each bar shows MAL. The red bars show data for the “PA stimuli”, where PA was increased for a fixed PPD (25 μs), and the blue bars show data for the “PPD stimuli”, where PPD was increased from 25 μs for a fixed PA. Note that there are missing data for some test ears due to electrode deactivation

**Fig. 3:**
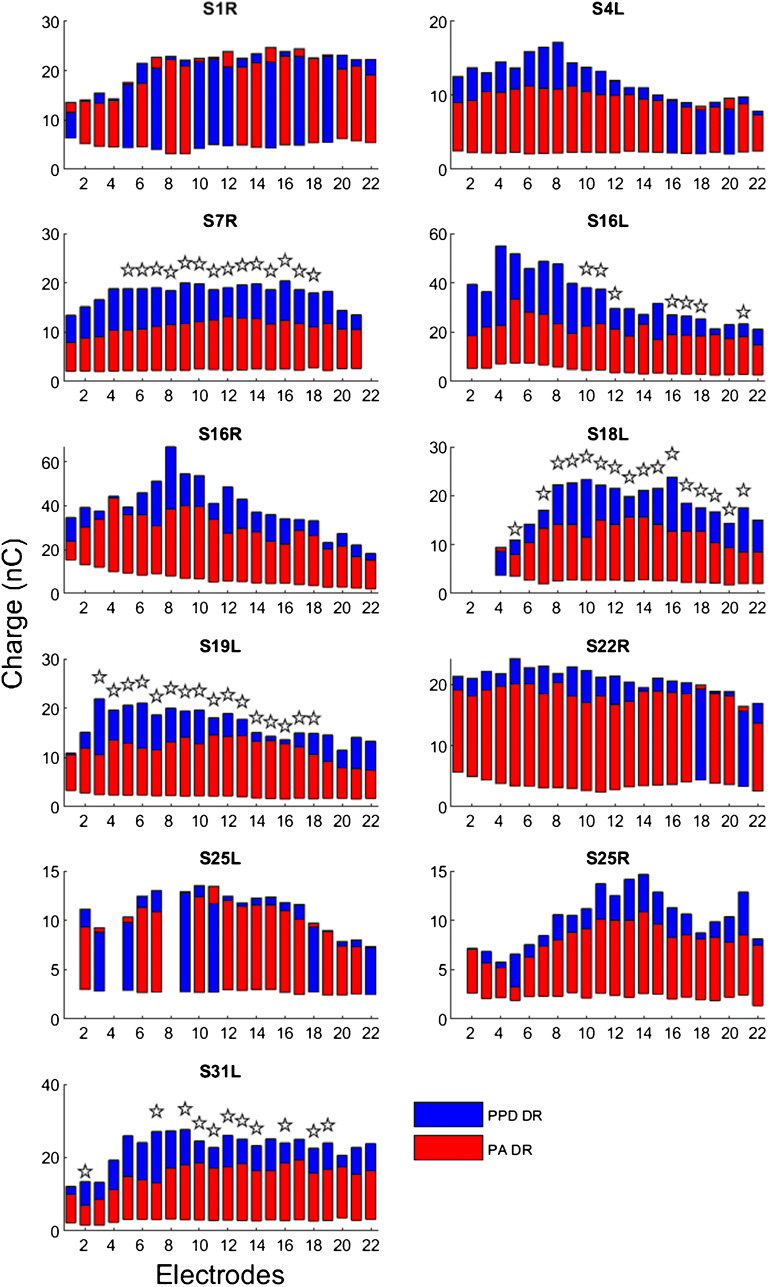
Same as Fig. 2, but with the 50-μs anchor. The open stars indicate electrode for which DR for the PPD stimuli could not be fully measured, due to hardware limitations that limited maximum PPD to 429.8 μs. Note that there are missing data for some test ears due to electrode deactivation

Figures 2 and 3 show DRs for the PA and PPD stimuli across electrodes for each test ear with the 25-μs and 50-μs anchors, respectively. As described in the “Methods” section, when measuring thresholds for the PPD stimulus, participants adjusted the PPD for the fixed PA to confirm that the 25 μs and 50 μs values indeed elicited threshold. The threshold data (in charge) were analyzed using a LMM, with stimulus type (PA, PPD) and anchor (25 μs, 50 μs) as fixed factors, and test ear as a random factor. Results showed no significant effect for stimulus type [*F*_(1, 914)_ = 1.6, *P* = 0.212], and there was no significant interaction between anchor and stimulus type [*F*_(1, 914)_ = 1.9, *P* = 0.165]. As there was no significant effect of stimulus type, thresholds (in nC) were averaged across the PA and PPD stimuli; these averaged thresholds are shown in Figures 2 and 3. For some electrodes in some test ears (S7R, S16L, S18L, S19L, S31L), MAL for the 50-μs anchor could not be achieved without exceeding the 429.8 μs PPD hardware limit (indicated by the stars in Fig. 3); these data were included in the analyses but under-estimated the PPD DR. Note also that in some cases, data were missing on deactivated electrodes.

The DR data were analyzed using a LMM, with stimulus type (PA, PPD), anchor (25 μs, 50 μs), and electrode as fixed factors, and test ear as a random factor. Results showed that DRs were significantly smaller for the PA than for the PPD stimulus [*F*_(1, 851)_ = 223.6, *P* < 0.001], and significantly smaller with the 25-μs anchor than with the 50-μs anchor [*F*_(1, 851)_ = 46.8, *P* < 0.001]; there was a significant interaction [*F*_(1, 851)_ = 23.3, *P* < 0.001]. PPD and PA DRs were also compared within test ears using paired t-tests. With the 25-μs anchor, PPD DRs were significantly larger than PA DRs for test ears S7R, S16L, S16R, S18L, and S19L (all *P* < 0.05). For S22R and S25L, PPD DRs were significantly smaller than PA DRs (both *P* < 0.05). PA and PPD DRs were not significantly different for the remaining test ears (all *P* > 0.05). With the 50-μs anchor, paired *t* tests showed that PPD DRs were significantly larger than PA DRs for all test ears (all *P* < 0.05), except for S1R. Paired t-tests on mean data (collapsed across electrodes) showed no significant difference in PA DRs between the 25-μs and 50-μs anchors (*t*_(10)_ = − 0.65, *P* = 0.530); however, PPD DRs were significantly larger with the 50-μs anchor than with the 25-μs anchor (*t*_(10)_ = -3.11, *P* = 0.010). There was a significant effect of electrode, which might represent local or regional influence on DRs [*F*_(21, 851)_ = 17.4, *P* < 0.001]; however, there was no significant interaction between electrode and anchor [*F*_(21, 851)_ = 0.1, *P* > 0.999] or stimulus type [*F*_(21, 851)_ = 0.8, *P* = 0.736]. With the 25-μs anchor, there was significantly greater across-site variance in PPD DRs than in PA DRs (*t*_(10)_ = 3.30, *P* = 0.007). As revealed by *F*-tests conducted on individual test ears, this difference was driven by two test ears (S19L: *F* (_21,21_) = 3.80, *P* < 0.05; S25L: *F* (_19,19_) = 2.54, *P* < 0.05). There was no significant difference in across-site variance between the PA and PPD DRs with the 50-μs anchor (*t*_(10)_ = 1.23, *P* = 0.245).

**Fig. 4:**
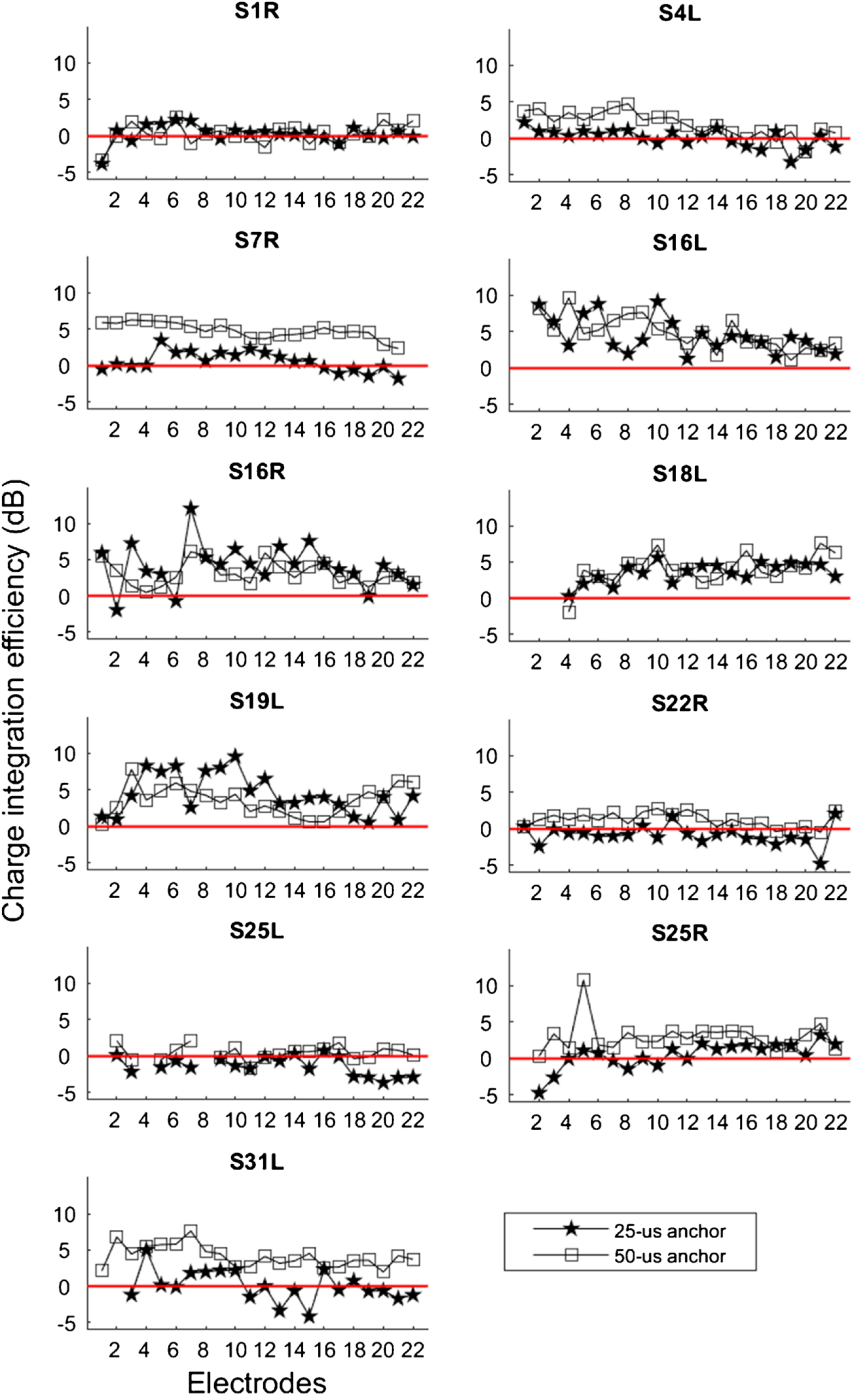
Charge integration efficiency (CIE) as a function of electrode for test ears with the 25-μs (filled stars) and the 50-μs anchors (open squares). The red line at zero represents the same DR with increasing PA or PPD. Values > 0 indicate poorer charge integration with increasing PPD (i.e., larger DRs with the PPD stimuli than with the PA stimuli)

Figure 4 shows CIE for each test ear with the 25-μs and 50-μs anchors; values greater than 0 suggest leakier charge integration. The CIE data were analyzed using a LMM, with anchor (25 μs, 50 μs) and electrode as fixed factors, and test ear as a random factor. Results showed that CIE was significantly smaller with the 25-μs anchor than with the 50-μs anchor [*F*_(1, 411)_ = 56.5, *P* < 0.001]. There was a significant effect of electrode [*F*_(21, 411)_ = 1.8, *P* = 0.018], but there was no significant interaction between electrode and anchor [*F*_(21,411)_ = 0.6, *P* = 0.908]. Across-site variance in CIE was compared between the 25-μs and 50-μs anchors within each test ear using *F* tests; in most cases, the variance did not significantly differ between the 25-μs and 50-μs anchors. For S18L, variance was greater with the 50-μs anchor (*F*_(21, 21)_ = 2.3905, *P* < 0.05). For S16R, variance was greater with the 25-μs anchor (*F*_(21, 21)_ = 3.43, *P* < 0.05). As shown in Figures 2 and 3, the common thresholds that anchored the PA and PPD DRs varied across electrodes and test ears. To determine whether threshold levels affected CIE, thresholds (in nC) and CIE were first normalized to their respective mean values within each test ear, thereby removing the variability across test ears. No significant correlation was observed between normalized thresholds and CIE for the 25-μs anchor (*r*_(230)_ = 0.001, *P* = 0.980) or the 50-μs anchor (*r*_(232)_ = 0.11, *P* = 0.110).

Stepwise multi-linear regression was performed to examine potential predictors for duration of profound hearing loss, defined as the duration between the onset of profound hearing loss and implantation. Eight predictors entered in stepwise order, including across-site mean in PA DR with the 25-μs and 50-μs anchors, across-site mean in PPD DR with the 25-μs and 50-μs anchors, across-site mean in CIE with the 25-μs and 50-μs anchors, and across-site variance in CIE with the 25-μs and 50-μs anchors. Across-site mean and variance measures were chosen as predictors based on the assumption that longer hearing deprivation may lead to generally poorer and/or more variable neural condition along the tonotopic axis. The stepwise regression was used to control for possible co-linearity among the predictors. Results showed that the across-site mean in PPD DR with the 50-μs anchor was the only significant predictor of duration of profound hearing loss (adjusted *r*^2^_(1)_ = 0.496; *P* = 0.009). Stepwise multi-linear regression was also performed between duration of any hearing loss (defined as when mild-to-moderate hearing loss was first reported) and the eight predictors described above. A model that included the across-site mean in CIE with the 25-μs and 50-μs anchors, as well as the across-site variance in CIE with the 50-μs anchor, nearly perfectly accounted for the variability in duration of any hearing loss across test ears (adjusted *r*^2^_(3)_ = 0.949; *P* < 0.001). Details of the regression statistics are shown in Table 2.

**Table 2:** Significant stepwise regression results comparing the across-site mean (ASM) or across-site variance (ASV) in PPD DR or CIE to duration of deafness or duration of hearing loss

	Unstandardized coefficients		Standardized coefficients		
	B	Std. Error	Beta	t	Sig.
Model for duration of deafness					
Constant	− 65.17	24.04		− 2.71	0.024
ASM PPD DR 50-μs anchor	3.33	1.01	0.739	3.29	0.009
Model statistics: *F*_*(1,9)*_ = 10.84, *P* = 0.009; *r*^*2*^ = 0.547; adjusted *r*^*2*^ = 0.496					
Model for duration of hearing loss					
Constant	25.45	2.24		11.35	<0.001
ASM CIE 25-μs anchor	5.53	0.59	1.06	9.41	<0.001
ASM CIE 50-μs anchor	2.15	0.64	0.31	3.35	0.012
ASV CIE 50-μs anchor	− 3.53	0.82	− 0.46	− 4.30	0.004
Model statistics: *F*_*(3,7)*_ *=* 62.41, *P* < 0.001; *r*^*2*^ = 0.964; adjusted *r*^*2*^ = 0.949					

**Fig. 5:**
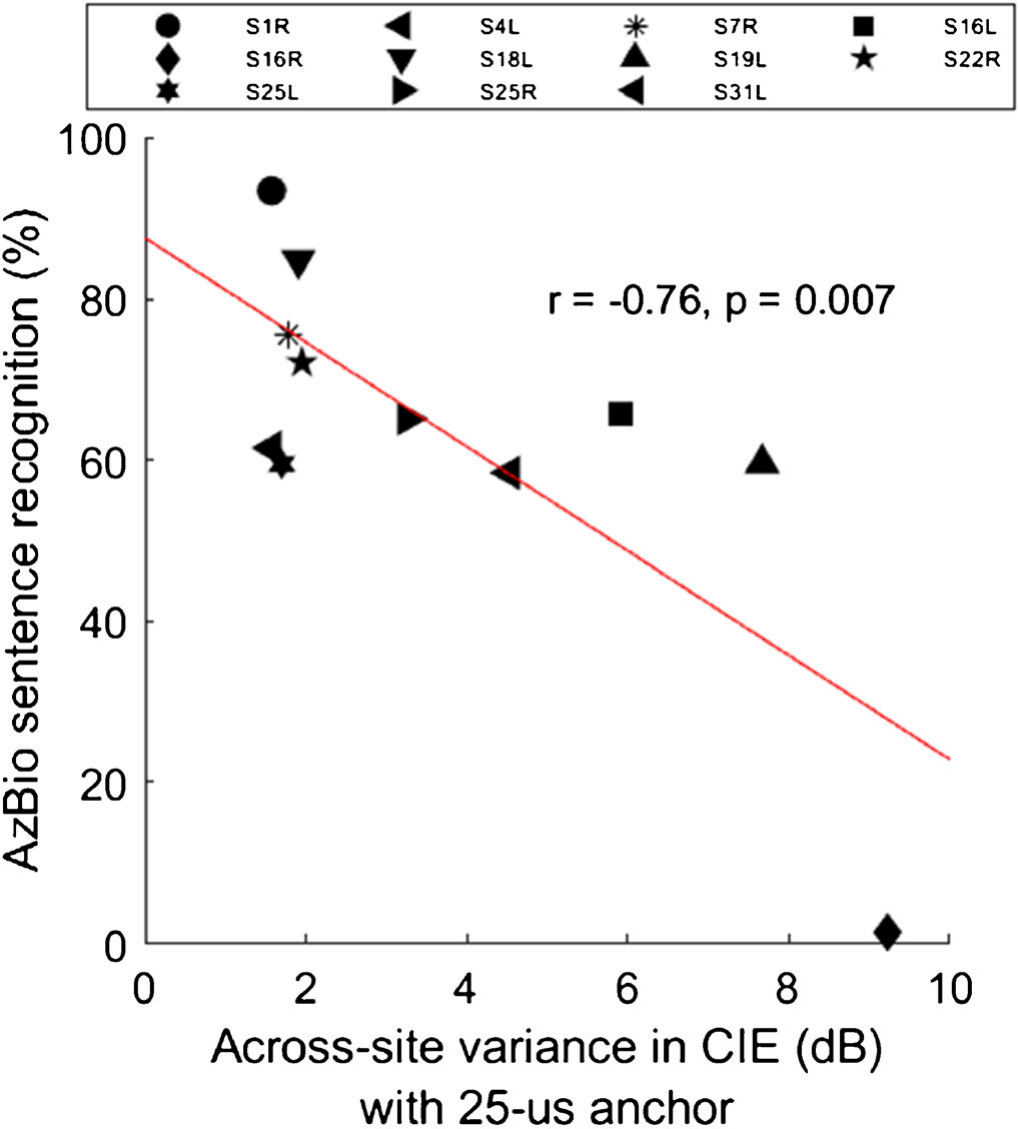
AzBio sentence recognition in 10-talker babble (20 dB SNR) as a function of the across-site variance in CIE with the 25-μs anchor. The red line shows the linear regression fit to the data, and *r* and *P* values are shown in the middle of the panel

Stepwise multi-linear regression was performed to identify predictors of speech recognition performance. The eight predictors described above were entered in the regression in a stepwise manner. The across-site variance in CIE with the 25-μs anchor was the only significant predictor of AzBio sentence recognition scores in 10-talker babble (*r*_(10)_ = − 0.76, *P* = 0.007; Fig. 5).

### Experiment 2: Strength-Duration Functions

**Fig. 6:**
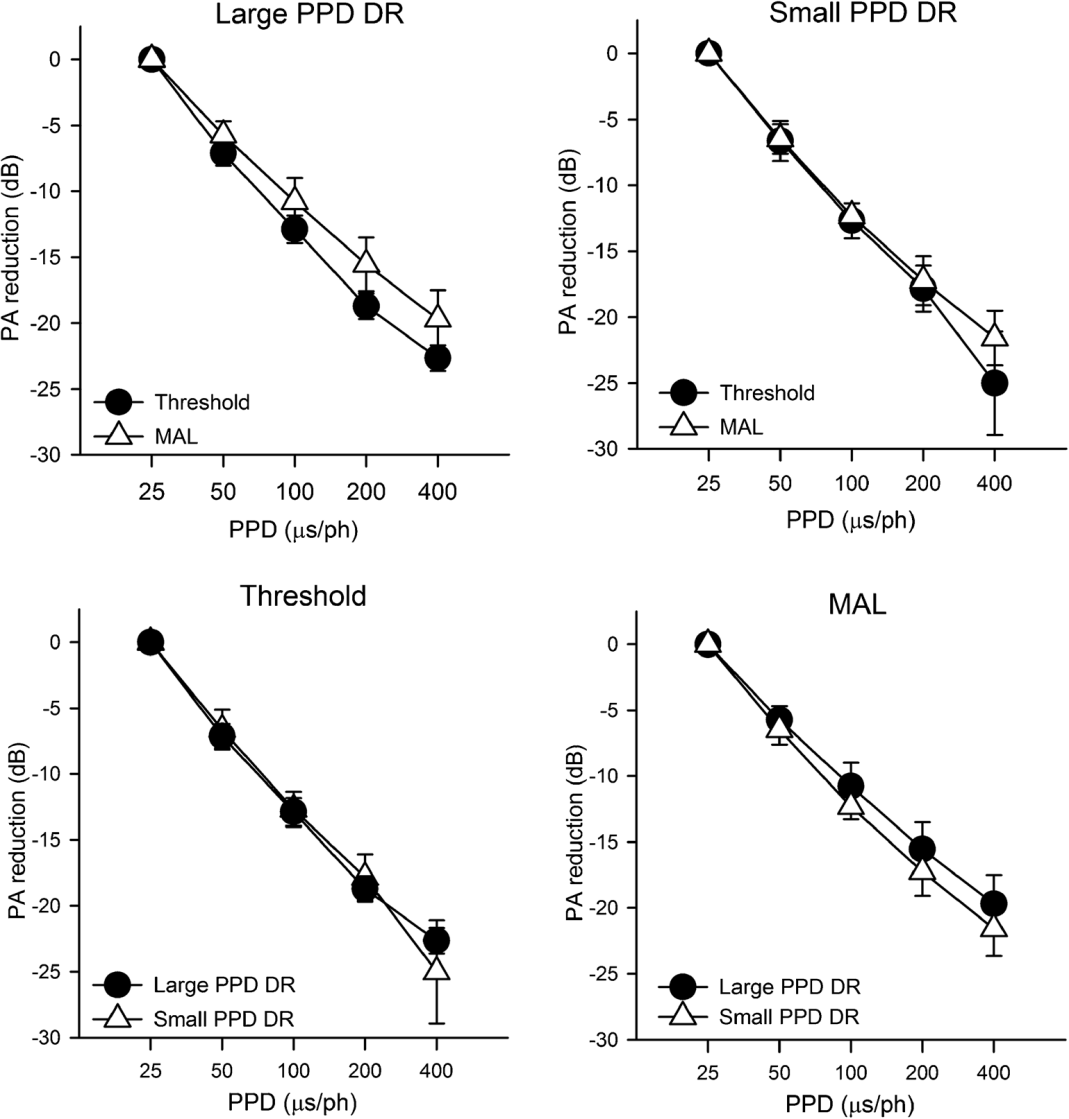
Top panels: Mean PA reduction at threshold (filled circles) and MAL (open triangle) as a function of PPD for electrodes with relatively large (left) or small PPD DRs (right). Bottom panels: Mean PA reduction with electrodes with relatively large (filled circles) or small PPD DRs (open triangles) as a function of PPD at threshold (left) or MAL (right). The error bars show the standard deviation

Strength-duration functions (the reduction in PA as a function of increasing PPD) are shown in Fig. 6. As described in the “Methods” section, two electrodes with relatively large PPD DRs or small PPD DRs with the 50-μs anchor were selected to assess strength-duration functions. The effects of measurement (thresholds versus MAL) are shown in the top panels of Fig. 6, while the effects of PPD DR size (large versus small) are shown in the bottom panels of Fig. 6. Note that thresholds could not be measured in most cases at 400 μs and in some cases at 200 μs because the PPD was too long, causing PA to reach device minimum while the stimulus remained audible (see Appendices 1 and 2 for raw data). The reduction in PA at threshold and MAL was calculated for each doubling in PPD (25–50, 50–100, 100–200, and 200–400 μs). A LMM was used to analyze the PA reduction data, with PPD doubling (25–50, 50–100, 100–200, 200–400 μs), PPD DR (large, small), and measurement (threshold, MAL) as fixed factors, and test ear as a random factor. Results showed a significant effect for PPD doubling (*F*_(3,131)_ = 22.1, *P* < 0.001) and for measurement (*F*_(1,131)_ = 14.6, *P* < 0.001), but not for PPD DR (*F*_(1,131)_ = 1.6, *P* = 0.203); there were no significant interactions. Post hoc Bonferroni pair-wise comparisons showed that the reduction in PA at threshold and MAL was significantly larger for the 25–50 μs range than for the 50–100, 100–200, and 200–400 μs ranges (*P* < 0.05 in all cases), significantly larger for the 50–100 μs range than for the 100–200 and 200–400 μs ranges (*P* < 0.05 in both cases).

**Fig. 7:**
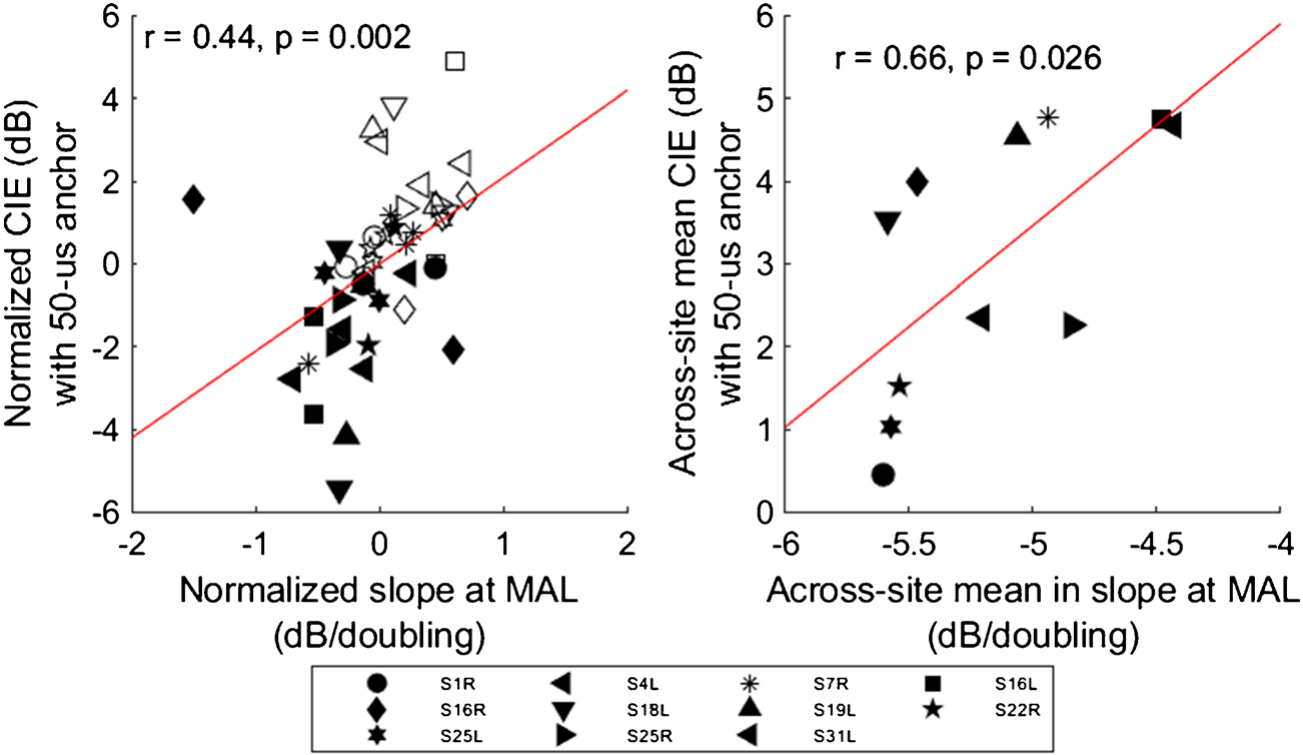
Left panel: Normalized CIE with the 50-μs anchor measured in Experiment 1 as a function of normalized strength-duration slopes at MAL. For each test ear, CIE and slopes were normalized to the across-site mean. Right panel: Across-site mean in CIE as a function of across-site mean in strength-duration slopes measured at MAL (dB/doubling of PPD). The red lines show linear regressions fit to the data, and *r* and *P* values are shown for each data set

Each strength-duration function was fit with a linear slope. At threshold, the mean slope across electrodes and test ears was − 6.18 ± 0.53 and − 6.12 ± 0.63 for electrodes with relatively large and small PPD DRs, respectively. At MAL, the mean slope across electrodes and test ears was − 4.92 ± 0.55 and − 5.39 ± 0.58 for electrodes with relatively large and small PPD DRs, respectively. The strength-duration slopes were compared to CIE with the 50-μs anchor measured in Experiment 1. Two comparisons were made. First, strength-duration slopes were compared to CIE across test electrodes, which required removing the across-ear variation in the two variables. To achieve this, the slopes and CIE values were normalized to their respective mean values within each test ear by subtracting the mean slope and the mean CIE from all data points within that ear. A significant correlation was observed between normalized slopes and CIE at MAL (*r*_(43)_ = 0.44, *P* = 0.002), but not at threshold (*r*_(43)_ = 0.16, *P* = 0.290), suggesting that steeper slopes at MAL were associated with better CIE. Second, the across-site means in strength-duration slopes and CIE were compared to examine whether the two variables were correlated across test ears. Again, a significant correlation was observed at MAL (*r*_(10)_ = 0.66, *P* = 0.026), but not at threshold (*r*_(10)_ = 0.33, *P* = 0.310). The significant within- and across-ear correlations are shown in Fig. 7.

The across-site mean of the strength-duration slopes was also compared to duration of hearing deprivation and speech recognition performance. No significant correlations were observed between duration of profound hearing loss and slopes at threshold (*r*_(10)_ = 0.32, *P* = 0.327) or MAL (*r*_(10)_ = 0.24, *P* = 0.473), or duration of any hearing loss and slopes at threshold (*r*_(10)_ = 0.33, *P* = 0.310) or MAL (*r*_(10)_ = 0.29, *P* = 0.380). No significant correlations were observed between speech recognition performance and slopes at threshold (*r*_(10)_ = − 0.44, *P* = 0.170) or MAL (*r*_(10)_ = − 0.04, *P* = 0.900). The standardized correlation coefficients were transformed to *Z* scores and the across-site mean in CIE was found to be the better predictor for duration of any hearing loss than strength-duration slopes at threshold (*z* = 2.37, *P* = 0.009) or MAL (*z* = 2.46, *P* = 0.007).

## DISCUSSION

The present study investigated sensitivity to PPD across electrodes and cochlear implant recipients. Sensitivity to PPD was evaluated using two methods: CIE and strength-duration functions. The correlation between these two measures, and their relationship to duration of hearing deprivation and speech recognition performance were examined.

Several observations regarding DR and the CIE measures are consistent with the idea that charge integration and the related excitation is less effective over relatively long PPDs than over short PPDs. First, PPD DRs were significantly larger with the 50-μs than the 25-μs anchor. Second, DRs were significantly larger (in terms of charge) when PPD was increased than when PA was increased, relative to a common threshold anchor; this effect was greater with the 50-μs anchor than with the 25-μs anchor. With the 50-μs anchor, all but one test ear exhibited larger DRs with increasing PPD than with increasing PA (CIE > 0 dB). With the 25-μs anchor, some test ears exhibited good charge integration (CIE ≈ 0 dB), with comparable across-site means between the PA and PPD DRs. Some test ears exhibited smaller PPD DRs than PA DRs at some electrodes (CIE < 0 dB), which is difficult to conceptualize. It is possible the neurons were driven less effectively with increasing PA than with increasing PPD; for example, there might be a stimulation site with relatively few neurons that have exceptional integration capabilities. It is also possible that the large PA DRs were due to less voltage being actually delivered than requested with high impedances. On average, PPD was increased to 134 μs from the 25-μs anchor to achieve full DR, and PPD was increased to 325 μs from the 50-μs anchor to achieve full DR (but sometimes incomplete in some test ears). However, using a longer PPD did not affect the excitability with increasing PA, as indicated by the comparable PA DRs with the 25-μs and 50-μs anchors. Previous studies that reported slower loudness growth with increasing PA used PPDs that were much longer than the 50 μs PPD used for the PA functions in the present study (Shannon 1985). Overall, the present data are consistent with previous findings that long PPDs are less effective in producing excitation than short PPDs, and this effect is more pronounced for longer PPDs.

The data in Figs. 2, 3, and 4 show that DRs and CIE varied considerably across electrodes for all test ears. This pattern of results was expected given the many previous psychophysical cochlear implant studies that report substantial across-site variance (Zhou and Pfingst 2016; Zhou et al. 2015, 2018, 2019). The across-site variance in CIE suggests that measurements using a single middle electrode (as in Zhou et al. 2020) may not be representative of the neural health condition of the whole array. One interesting observation is that PPD DRs were more variable across the electrode array than were PA DRs. This is presumably due to the increased current spread associated with increasing PA, resulting in broader recruitment and similar PA DRs on neighboring electrodes. With monopolar stimulation, at equal loudness, Zhou et al. (2020) found that relatively longer PPDs produced slightly but significantly less spread of excitation than did relatively short PPDs, suggesting that the PA and PPD DRs may reflect excitation of different but presumably overlapping neural populations. It is possible that this would add some noise and reduce place specificity in the variability of CIE measures across electrodes, but the issue may be resolved by using focused stimulation (e.g., Bierer 2007; Bierer and Litvak 2016). For most test ears, the across-site variance in CIE was comparable between the 25-μs and 50-μs threshold anchors. This was contrary to the expectation that the longer PPD anchor would result in potentially greater differentiation of leakiness across stimulation sites. However, PPD DRs were underestimated on some electrodes in some test ears with the 50-μs anchor due to hardware limitations, which may have reduced the across-site variance in the PPD DR and CIE measures.

The data also show that CIE was unrelated to specific electrodes and cochlear regions. Previous psychophysical and physiological studies have found an apical advantage for phase locking in the inferior colliculus (Middlebrooks and Snyder 2010), stimulus detection and gap detection thresholds (Bierer et al. 2015), and ECAP amplitude growth (Brill et al. 2009). Such an apical advantage might be expected in individuals with progressive sensorineural hearing loss, since neural degeneration typically begins at the basal region. In the present study, there was no evidence of an apical advantage (or any systematic advantage for any cochlear region) for CIE, possibly due to individual differences in insertion depth. Consistent with Zhou et al. (2020), the present CIE data (with a larger sample size and all available electrodes) did not depend on the level of the threshold anchor. The present data support the notion that CIE may be robust to other factors contributing to variation in detection thresholds such as electrode placement.

There was no relationship between CIE and duration of profound hearing loss, in contrast to our previous findings (Zhou et al. 2020). Note that across-site data were used to predict duration of profound hearing loss in the present study, while CIE was measured on a single electrode in Zhou et al. (2020). We further quantified hearing deprivation in terms of duration of any hearing loss, based on the assumption that neural degeneration may begin as soon as hearing loss was identified, long before the onset of severe-to-profound deafness. Results showed that when combined, the across-site variance and across-site mean in CIE almost perfectly accounted for the variability in duration of any hearing loss across test ears (*R*^*2*^ =0.94). Note that PA DR, which is most commonly used for clinical mapping, was not correlated with duration of profound hearing loss or duration of any hearing loss. It is interesting that not only the across-site mean, but also the variation in CIE accounted for duration of any hearing loss, with greater variation associated with longer hearing deprivation. These results suggest that there might be an increase in the patchiness of neural condition as hearing loss progresses. Interestingly, the across-site variance in CIE was also a strong predictor for speech recognition performance. Note that speech performance was measured using the clinical speech processor settings (e.g., adaptive dynamic range optimization, directional microphones, etc.), which may have differed among test ears and resulted in some variability in speech measures. However, it is unlikely that the potential variability in speech performance due to differences in clinical settings would predict the across-site variability in CIE. Taken together, these correlations suggest that longer hearing deprivation results in greater and more variable degeneration across the array, as well as poorer speech recognition outcomes.

While CIE appears to be a promising behavioral measure with which to probe neural degeneration in humans, there are several caveats to consider. First, duration of hearing deprivation is not the strongest predictor of neural degeneration. Nadol et al. (1989) found that etiology of hearing loss accounted for 59 % of the variance in spiral ganglion counts in deafened ears. Thus, the extent to which CIE characterizes neural degeneration may depend on the characteristics of the subject sample. In the present study, etiology of deafness was hereditary in 8 out of 11 test ears (with the source of hereditary hearing loss known in only 3 test ears), suggesting that etiology was not a strong predictor of the CIE data. Second, some previous research suggests that the effect of electrode distance may differ across phase durations (e.g., McKay and McDermott 1999). Therefore, the effect of electrode position may not be the same for the PA and PPD DRs, and may contribute to the CIE measure. Third, as discussed above, the smoother PA DR (across the array) suggests spectral smearing due to the increasing current spread associated with increasing PA, which may limit the electrode specificity and the across-site variance in CIE measures.

Previous physiological studies provide strong evidence that sensitivity to PPD measured using strength-duration functions depends on neural degeneration (Van den Honert and Stypulkowski 1984; Shepherd et al. 2001). The characteristic of the strength-duration function that relates to the time constant of the target neural membrane is termed “chronaxie,” which is proportional to the product of membrane resistance and capacitance, and is defined as the duration (i.e., the PPD) at a current level that is twice the threshold as PPD approaches infinity. Smaller chronaxie (i.e., shorter time constants) indicates leakier integration. Van den Honert and Stypulkowski (1984) reported longer chronaxie in single fibers for normal-hearing animals than for animals whose peripheral processes had been mechanically removed. Similarly, Shepherd et al. (2001) reported longer chronaxie in animals that were acutely deafened than in those that were deafened over a longer term. The acutely deafened animals exhibited complete loss of hair cells but intact auditory nerve, while the long-term deafened animals had substantial cell loss and degenerated peripheral processes in the surviving cells. The shorter chronaxie was mainly explained by the loss of peripheral processes and activation of the extensively myelinated (low capacitance) central axons. Note that theses animal studies allowed for use of longer PPDs than would be possible for studies with humans to derive chronaxie. For compound neural responses such as ECAPs, previous studies have shown that the dependence of PPD sensitivity on neural survival is more apparent with longer PPDs (Prado-Guitierrez et al. 2006; Ramekers et al. 2014).

In the present study, strength-duration functions were measured at selected electrodes with relatively large and small PPD DRs, and were compared to CIE measures. Regardless of the electrodes, there was greater reduction in PA for the smaller doubling in PPD (25–100 μs) than for the larger doubling in PPD (100–400 μs), and slopes were steeper at threshold than at MAL. The strength-duration data agreed with the CIE data, in that both measures revealed greater leakiness for larger PPDs. The strength-duration slopes were correlated with CIE across electrodes within and across test ears, but did not predict duration of hearing deprivation or speech recognition performance. As such, the strength-duration data are puzzling. One possibility is that, unlike single fiber recordings, steep behavioral strength-duration functions might reflect relatively broad stimulation, (e.g., a lateral wall electrode that is spatially distant from neurons) rather than better neural health. This is supported by previous psychophysical studies that showed steeper strength-duration functions with monopolar than with bipolar stimulation (Miller et al. 1999; Smith and Finley 1997). It is also possible that measuring strength-duration functions at only four electrodes did not adequately represent the condition of the whole cochlea. Alternatively, comparing loudness growth with increasing PA or PPD in terms of charge, rather than the strength-duration slopes which represent the tradeoff between PA and PPD (as in Zeng et al. 1998; Chatterjee et al. 2000; Chatterjee and Kulkarni 2014), may be more sensitive to neural health and implant function in implant recipients. The present method for calculating charge integration also contextualizes neural leakiness within clinically relevant DRs. Future studies are warranted to disentangle the potential contribution of non-neural factors to PPD sensitivity (e.g., the proximity of electrodes to neural populations).

## Appendix

**Appendix 1:** PA levels for each PPD and slope across PPDs at threshold for electrodes (EL) with relatively large and small PPD DRs measured with the 50-ms anchor in Experiment 1.

Test ear	Threshold (dB re 1 A)													
	Sites with larger PPD DR							Sites with smaller PPD DR						
	EL	25 s/ph	50 s/ph	100 s/ph	200 s/ph	400 s/ph	slope (dB/doubling)	EL	25 s/ph	50 s/ph	100 s/ph	200 s/ph	400 s/ph	slope (dB/doubling)
S1R	8	43.22	36.39	29.12	23.42		-6.67	2	46.48	40.99	34.66	28.3	24.24	-5.72
	14	45.03	38.94	32.07	25.22		-6.63	4	44.45	38.44	32.55	27.1		-5.80
S4L	7	36.21	29.03	25.66			-5.28	18	38.94	29.91	23.69			-7.62
	8	38.62	30.35	24.93			-6.85	22	37.89	32.55	25.75	20.34		-5.94
S7R	9	34.84	27.36				-7.48	1	34.57	27.95	23.14			-5.72
	16	36.74	28.66	25.28			-5.73	21	37.8	30.25	24.94			-6.43
S16L	4	48.06	42.49	35.83	31.08	25.89	-5.57	19	41.9	31.58	28.56			-6.67
	5	49.94	43.34	37.04	32.55	25.83	-5.90	22	39.64	33.4	28.5	23.08		-5.46
S16R	8	49.88	43.34	38.18	31.87	27.92	-5.54	1	55.45	47.83	41.11	34.54	27.65	-6.89
	9	49.03	42.2	36.54	30.67	26.66	-5.63	22	37.53	34.1	23.37	22.11		-5.70
S18L	8	38.85	32.73	26.01	20.21		-6.27	4	44.78	37.33	30.26	24.63		-6.75
	10	41.05	34.1	27.92	21.03		-6.63	5	41.2	36.92	28.47	23.96		-6.02
S19L	3	38.44	31.61	26.13	20.12		-6.16	1	41.81	34.28	28.59	22.17		-6.46
	6	36.62	30.56	25.63			-5.50	20	35.75	28.39	23.43			-6.16
S22R	5	42.68	35.31	29.78	24.1		-6.13	21	42.8	35.33	29.35	24.78		-6.00
	7	42.03	35.85	28.31	23.53		-6.30	22	40.86	34.34	29.6	23.06		-5.81
S25L	7	40.44	32.61	27.24	21.32		-6.27	21	38.3	32.61	26.71			-5.79
	10	40.11	33.25	26.14	21.64		-6.25	22	39.35	31.79	25.6	21.06		-6.11
S25R	13	39.12	29.69	25.4	21.33		-5.76	2	40.22	32.93	26.13			-7.04
	14	40.14	32.65	26.79	20.91		-6.36	4	37.4	32.26	24.56			-6.42
S31L	7	43.69	36.23	29.19	25.32		-6.21	1	37.49	31.6	26.83	22.8		-4.88
	9	43.68	35.19	29.75	23.01		-6.75	3	34.56	28.8	24.21			-5.18

**Appendix 2:** PA levels for each PPD and slope across PPDs at maximum acceptable loudness (MAL) for electrodes (EL) with relatively large and small PPD DRs measured with the 50-ms anchor in Experiment 1.

Test ear	MAL (dB re 1 A)													
	Sites with larger PPD DR							Sites with smaller PPD DR						
	EL	25 s/ph	50 s/ph	100 s/ph	200 s/ph	400 s/ph	slope (dB/doubling)	EL	25 s/ph	50 s/ph	100 s/ph	200 s/ph	400 s/ph	slope (dB/doubling)
S1R	8	58.53	52.37	47.09	39.35	35.66	-5.88	2	54.31	49.2	42.17	37.59	31.44	-5.74
	14	58.18	51.84	45.51	41.11	35.31	-5.65	4	53.43	48.5	42.87	38.12	32.84	-5.15
S4L	7	53.25	48.15	42.87	38.47	33.72	-4.87	18	52.37	44.98	38.65	33.72	28.44	-5.91
	8	52.37	47.27	42.87	38.47	34.07	-4.54	22	50.08	45.33	37.94	33.19	28.62	-5.51
S7R	9	53.6	47.27	42.52	37.77	35.04	-4.66	1	50.87	44.63	39.7	35.57	31.17	-4.85
	16	54.31	48.76	42.17	38.47	35.83	-4.72	21	54.04	47	41.64	36.54	31.7	-5.52
S16L	4	61.34	59.06	53.78	50.61	46.21	-3.87	19	62.4	52.72	49.03	44.45	41.46	-5.01
	5	62.75	58.7	56.42	50.79	46.56	-4.03	22	57.12	52.02	46.74	41.99	37.06	-5.01
S16R	8	61.17	55.89	53.95	46.39	42.17	-4.75	1	61.34	55.01	48.32	38.82	34.6	-6.97
	9	64.16	57.82	52.55	48.15	42.69	-5.26	22	55.71	50.08	44.28	39.7	36.54	-4.87
S18L	8	56.59	50.26	44.45	40.94	36.01	-5.05	4	53.43	46.39	39.7	34.07	30.03	-5.91
	10	57.82	51.67	46.39	40.23	36.19	-5.47	5	54.31	48.15	41.99	37.59	30.03	-5.91
S19L	3	52.19	45.33	39.7	35.66	31.44	-5.12	1	51.84	45.68	40.41	34.6	30.73	-5.33
	6	52.9	46.74	41.81	38.3	34.07	-4.61	20	50.61	44.1	38.65	33.37	30.03	-5.19
S22R	5	58.88	52.02	46.39	40.58	36.54	-5.61	21	57.65	50.26	44.1	39.35	34.95	-5.63
	7	58.18	51.84	46.56	40.58	36.36	-5.49	22	56.42	50.26	43.75	38.65	35.13	-5.42
S25L	7	52.9	46.56	40.94	36.36	32.67	-5.07	21	49.91	42.52	36.71	29.85	26.16	-6.02
	10	54.31	47.44	41.99	36.54	31.61	-5.63	22	48.32	41.81	34.95	30.73	25.98	-5.58
S25R	13	51.49	46.21	41.64	37.77	33.9	-4.36	2	49.03	43.05	37.24	32.14	28.44	-5.21
	14	52.37	47.09	41.99	37.94	33.72	-4.64	4	47.44	41.64	34.95	30.91	27.04	-5.15
S31L	7	54.48	49.38	42.69	40.58	36.71	-4.43	1	50.08	42.17	38.3	34.6	31.08	-4.56
	9	55.18	49.38	45.51	39.88	37.24	-4.54	3	50.79	43.57	38.3	36.71	33.19	-4.20
